# Exploring Headaches in Pediatric Behçet Disease: Prevalence, Clinical Impact, and Management

**DOI:** 10.3390/jcm13133659

**Published:** 2024-06-23

**Authors:** Andrea Santangelo, Antonio Corsello, Gilda Gizzi, Maddalena Lancieri, Maria Cristina Diana, Federica Trucco, Alessandro Orsini, Alice Bonuccelli, Diego Giampietro Peroni, Lorenzo Perilli, Edvige Correnti, Giuseppe Santangelo, Pasquale Striano, Vincenzo Raieli

**Affiliations:** 1 Department of Neurosciences, Rehabilitation, Ophthalmology, Genetics, Maternal and Child Health (DINOGMI), University of Genoa, 16100 Genoa, Italy; pstriano@unige.it; 2 Pediatric Neurology, Pediatric Department, AOUP Santa Chiara Hospital, 56126 Pisa, Italyal.bonuccelli@gmail.com (A.B.); diego.peroni@unipi.it (D.G.P.); 3 Department of Clinical Science and Community Health, University of Milan, 20122 Milan, Italy; 4 Pediatric Neurology and Muscular Diseases Unit, Pediatric Department, IRCCS Istituto G. Gaslini, 16100 Genoa, Italy; 5 Clinical Paediatrics, Department of Molecular Medicine and Development, University of Siena, Azienda Ospedaliero-Universitaria Senese, 53100 Siena, Italy; 6 Child Neurology and Psychiatry Unit—ISMEP, “G. Di Cristina” Children’s Hospital—ARNAS Civico, 90127 Palermo, Italy; edvige.correnti@arnascivico.it (E.C.); giuseppe.santangelo@arnascivico.it (G.S.); vincenzoraieli@gmail.com (V.R.)

**Keywords:** Behçet’s disease, pediatric Behçet syndrome, headache, neuro-Behcet, migraine, vasculitis

## Abstract

Behçet’s Disease (BD), also recognized as Behçet Syndrome, manifests uniquely in pediatric populations as Pediatric Behçet’s Disease (PBD), characterized by multisystemic inflammatory symptoms including recurrent oral and genital aphthae, and diverse ocular, vascular, and neurological involvements. This review elucidates the prevalence, burden, and management strategies of headaches in children with PBD, focusing on both primary headaches, such as migraine and tension-type headaches, and secondary headaches linked to systemic disease manifestations. It explores the pathophysiological underpinnings specific to PBD-related headaches and discusses the intricate relationship between systemic inflammatory processes and neurological symptoms. By examining the literature from 2004 to 2024, this study highlights the high frequency of headache in PBD patients, underscoring its diagnostic and clinical significance. We aim to provide a detailed understanding of headache management in PBD, emphasizing tailored therapeutic strategies that address the unique challenges faced by this patient population. This review also underscores the importance of comprehensive clinical evaluations to optimize outcomes and mitigate long-term sequelae, proposing that awareness and understanding of headache in PBD can significantly enhance both diagnosis and management.

## 1. Introduction

Behçet’s Disease (BD), also known as Behçet Syndrome, first described in 1937 by Hulusi Behçet, is a systemic auto-inflammatory disorder classically characterized by oral aphthae, which can eventually be associated with genital aphthae (bipolar aphthosis), as well as a wide range of systemic symptoms, including ocular, vascular, gastrointestinal, neurological, and neuropsychiatric involvement.

The underlying pathophysiology is primarily linked to a vasculitic process potentially targeting both arterial and venous vessels of different calibers, a unique characteristic distinguishing BD from other systemic vasculitis. Indeed, BD is classified as a variable-vessel vasculitis in the 2012 Revised International Chapel Hill Consensus Conference Nomenclature of Vasculitides [1].

Genetic factors, particularly HLA-B5101 [2] and polymorphisms in genes associated with pro-inflammatory interleukins [3,4], have been linked to individual susceptibility to the development of BD. Environmental triggers such as infectious agents may also contribute to disease development [5,6].

Despite typically emerging in young adulthood with a prevalence of 10.3/100,000 [7], approximately 15–20% of all Behçet patients develop symptoms during childhood [8,9]. The term Pediatric Behçet’s Disease (PBD) has been therefore adopted to characterize the subset of patients whose onset of symptoms occurred prior to reaching 16 years of age. The mean age at pediatric onset ranges from 4.9 to 12.3 years, with an average delay in diagnosis of about 3 years [8,10].

As there is no specific laboratory test for the disease, the diagnosis of PBD is based on clinical criteria proposed in 2015 [11] and including at least three of the following:

- Recurrent oral aphthosis occurring at least three times annually;
- Genital ulceration or aphthosis, usually resulting in scarring;
- Skin involvement, characterized by necrotic folliculitis, acne-like lesions, or erythema nodosum;
- Ocular involvement, including anterior uveitis, posterior uveitis, and retinal vasculitis;
- Neurological signs, excluding isolated headaches;
- Vascular signs such as venous thrombosis, arterial thrombosis, or arterial aneurysm.

Neurological involvement, termed Neuro-Behçet’s Disease (NBD), occurs in 3.6 to 59.6% of pediatric cases, a prevalence possibly slightly higher than in adults [12], with headache representing the most frequent clinical symptom. However, headache, commonly characterized as pain localized above the orbitomeatal line, is one of the most prevalent complaints among children and adolescents, assuming a leading role in the development of disability within this age group. Primary headaches most commonly encountered in children include migraine and tension-type headache (TTH). TTH is often reported as the predominant type of primary headache in childhood. However, TTH is frequently diagnosed when the criteria for migraine are not met, which may lead to an overestimation of the prevalence of TTH and an underestimation of the prevalence of migraine [13].

The association between headaches and specific rheumatological conditions is of particular interest, possibly pointing at the existence of an intricate interplay between systemic diseases and neurological symptoms. Interestingly, recent studies have highlighted a high prevalence of migraine (29.1%) and TTH (13.8%) among pediatric patients diagnosed with Juvenile Idiopathic Arthritis and Familiar Mediterranean Fever [14]. Of note, in the context of BD, headaches emerge as a significant clinical indicator. In the PEDBD study, the presence of headaches exhibited a significant association with BD (*p* = 0.0063), underscoring its diagnostic relevance [11]. It is noteworthy that the prevalence of NBD among children demonstrates a range of 15 to 30%, where isolated headaches serve as the primary symptom in as many as 25% of documented cases. This phenomenon contributes to elevating the overall occurrence rate to 50%. [15].

However, due to the striking complexity of NBD pathophysiology, the evaluation and attribution of headache to the different potential underlying causes can be particularly challenging in these patients, leading to a considerable heterogeneity in available studies. The aim of this review is to shed light on the prevalence and clinical impact of pediatric headaches, trying to give a comprehensive evaluation of possible management strategies, particularly in the context of associated systemic conditions, for optimizing clinical outcomes and mitigating long-term sequelae.

## 2. Methods and Results

We performed a narrative literature review by performing an extensive search on the PubMed (Medline) database, covering publications from January 2004 to January 2024. This search, conducted on 1 February 2024, utilized the search terms ((behcet) AND (headache)) OR (Neurobehcet), and was limited to articles published in English. Following an initial screening of abstracts, two authors closely examined the full texts of the articles, selecting those pertinent for an in-depth analysis. The selected articles were then critically reviewed and included in our study. A total of 449 articles were initially identified. Out of these, 406 articles were excluded due to the unavailability of the full text and a lack of inclusion of pediatric patients. Consequently, 43 articles were deemed suitable and were included in our review.

A flowchart outlining the article selection and exclusion process is available in the Supplementary Materials.

## 3. Discussion

Headaches in children are a common issue that can significantly impact their quality of life and academic performance. While most headaches in children are not due to serious underlying causes, they can still have a detrimental effect on daily life.

The source of headache pain typically arises from structures surrounding the brain, such as blood vessels, meninges, muscle fibers, facial structures, and cranial or spinal nerves. These structures contain nociceptors that, when stimulated by factors like vascular changes, produce a pro-inflammatory response leading to pain perception [16]. The distribution of pain can be unilateral or bilateral depending on the innervation patterns of trigeminal, vagus, or glossopharyngeal nociceptors [17]. The pathophysiology of primary headaches, however, is not fully understood, as they can be triggered by different pathophysiological processes. It has been hypothesized that the onset of migraine could involve hypothalamic activation and the subsequent secretion of pituitary adenylate cyclase-activating polypeptide (PACAP), leading to vasodilation [18].

Environmental factors play a significant role in triggering headaches, particularly migraines, and can contribute to the progression from episodic to chronic migraines, especially in genetically predisposed individuals. Stress, sleep disturbances, hormonal changes, weather patterns, dietary factors, and sensory stimuli are among the critical triggers [19]. Additionally, noise exposure has been linked to increased pain severity and decreased tolerance to aversive stimuli in headache patients [20].

While secondary headaches in children are rare, they necessitate thorough evaluation. Genetic contributions to childhood migraines, except in specific cases like hemiplegic migraine, remain incompletely understood [21].

### 3.1. Primary Headaches in BD

Primary headaches represent the most prevalent complaints among the pediatric population, significantly impacting physical and psychological well-being. Migraine and TTH rank as the most common types, followed by chronic headaches and probable medication-overuse headaches [13,19,20,21,22,23].

The actual incidence of these headaches may be underestimated due to inconsistent diagnostic standards and practices. Interestingly, the literature shows that headache incidence peaks at 13 years of age and that migraine rates are similar between boys and girls under the age of 10, although it increases in females during adolescence, likely due to hormonal changes [13].

Various theories have been proposed to explain migraine pathophysiology, including vascular, neural, and trigemino-vascular mechanisms. Migraines are thought to be triggered by changes in cerebral blood flow mediated by serotonin and neurogenic inflammation caused by trigeminal nerve activation [21].

Furthermore, some studies have demonstrated a bidirectional association between the central nervous system and the gastrointestinal tract, known as the gut–brain axis. Such an axis, modulated by immunological, hormonal, and neural pathways and gut microbiota, may trigger migraine attacks through increased CGRP secretion and pro-inflammatory factors, particularly in response to microbiome changes induced by stress or antibiotic therapy [22,23].

Some studies have shown a linkage between immunological and neurological conditions, such as migraine. In particular, the etiology of autoimmune disorders may entail genetic factors encoding for human leukocyte antigens (HLAs) and cytokines. Notably, variants within HLA class I genes have been correlated with an increased predisposition to migraine [24,25]. These findings suggest a common genetic background between migraine and immunological disorders. It is known that in the pathogenesis of BD, pro-coagulant factors such as fibrinogen, thrombin, and protein C may have a role in causing systemic inflammation and thrombus formation, and are thus responsible for the perception of pain. Regarding headache in BD, an intense inflammatory response associated with a huge expression of pro-inflammatory cytokines can contribute to the sensitization of trigeminal nociceptors, although there is a lack of data on such an aspect [26].

In children and adolescents, migraine is the predominant primary headache, characterized by specific criteria including duration, location, quality, intensity, and accompanying symptoms. Diagnosis requires at least five attacks meeting certain criteria, with migraine without aura being the most common pattern in children, typically featuring frontal, pounding headaches lasting 2 to 72 h. TTH is also prevalent, characterized by mild-to-moderate pressing or constricting pain without associated symptoms like nausea or photophobia. Pediatric headache conditions are often overlooked or misdiagnosed, posing challenges for accurate diagnosis.

Migraines are associated with various comorbidities such as allergies, sleep disorders, emotional and behavioral problems, depression, and anxiety, all of which can impact treatment outcomes. Among patients with BD, primary headaches, especially migraine or TTH, are common. Recurrence of symptoms is often linked to the exacerbation of systemic symptoms, primarily oral ulcers, with accompanying mild-to-moderate pain and symptoms like nausea, vomiting, or photophobia [27].

Primary headaches are the most common type of headache in patients with BD, and the majority of patients develop these symptoms after the initial diagnosis of BD.

Interestingly, there is a significant disparity in percentages among studies. Saip et al. reported a higher prevalence of TTH compared to migraine (23.6% vs. 14.9%) in a cohort of patients with an average age of 36 ± 9 years, with a higher prevalence in women [28]. Similarly, Moghaddassi et al. confirmed this finding, reporting a prevalence of TTH at 12.2%, compared to 9.1% for migraine in an adult population with an average age of 39.6 years [29]. Conversely, Aykutlu et al. reported a higher prevalence of migraine compared to TTH in a slightly younger cohort (26.9 ± 9.8 years) [30]. Concerning children, primary headaches in pediatric NBD have been reported anecdotally [31].

### 3.2. Secondary Headaches in BD

NBD is generally regarded as comprising two clinically and pathophysiologically distinct entities: parenchymal and non-parenchymal NBD (npNBD). Parenchymal NBD (pNBD) defines the inflammatory involvement of the brain—the central counterpart of the peripheral vascular immune manifestation of the disease—whereas npNBD corresponds to the damage resulting from pathological processes—mainly vaso-occlusive events—occurring in the brain. Importantly, headache can be a symptom of both forms of NBD.

Based on the studies reviewed (Table 1 and Table 2), we found that 14 out of 54 pediatric patients (26%) experienced secondary headaches as a component of their pNBD symptoms. Amidst this group, only 1 out of 14 (7%) showed isolated meningeal involvement [32], whereas the remaining 13 (93%) had a variable degree of central parenchymal lesions [12,33,34,35,36,37,38,39,40]. Considering pNBD onset, aside from headache, it included sub-acute symptoms of cerebral involvement—including pyramidal signs, pseudobulbar paralysis, hemiplegia, cranial nerve palsies, and seizure, depending on the affected area of the brain. Fever was described at the onset in two cases of rhombencephalitis [32,38] and in an NBD patient presenting with acute disseminated encephalomyelitis (ADEM) [37]. Interestingly, in the majority of cases (7 out of 11), neurological symptoms appeared prior to BD diagnosis. Nevertheless, some reported a prior medical history of recurrent aphthosis.

When evaluated at MRI, pNBD presented with hyperintense T2 lesions involving mainly brainstem, thalami, internal capsule, and to a lesser extent also the basal ganglia, spinal cord, and cortex. Patients with a presentation of meningoencephalitis underwent a lumbar puncture, which showed sterile CSF pleocytosis and increased protidorrachia. Treatment included, in almost all outlined cases, a combination of steroids, colchicine, and immunosuppressants (mainly azathioprine). Usually, patients affected by pNBD eventually develop a progressive disease course, with a worse outcome compared to the non-parenchymal subgroup.

Among npNBD cases, 40/54 (74%) patients in our query showing headache were found to have non-parenchymal neuro-Behçet disease. Notably, among these, 90% (36 cases) displayed cerebral sinus thrombosis [12,34,39,40,41,42,43,44,45]. Furthermore, other non-parenchymal forms of NBD observed included isolated intracranial hypertension (four cases, 10%) [41,46,47], focal demyelination [48] and non-specified forms (one case) [33]. As compared to the parenchymal NB group, a higher percentage (75%) of those affected by non-parenchymal disease already had a diagnosis of BD. Remarkably, different patients were stated to have symptoms of pseudotumor cerebri, presenting with either vomiting, blurred vision or diplopia, or a combination of these symptoms. Blurred vision and diplopia were mainly the result of increased intracranial pressure compressing the VI cranial nerve.

Interestingly, the cases with isolated intracranial hypertension had normal MRI cerebral imaging, and intracranial hypertension was disclosed indirectly by papilledema and confirmed by increased CSF opening pressure. Generally, biochemical and cultural cerebral fluid analyses yielded normal results. On the contrary, patients with central venous sinus thrombosis (CVST) usually had positive contrast-enhanced MRI or MRA showing thrombosis most commonly in either transverse sinus, but also in the rectal and superior sagittal sinuses. Additionally, patients affected by npNBD also received a combined treatment including intravenous or oral steroids, colchicine, and azathioprine with anticoagulant agents in cases of CVST and dehydrating drugs for CSF hypertension.

### 3.3. Clinical Management

The primary objectives in managing PBD encompass effectively mitigating inflammatory episodes and preventing enduring organ impairment. Treatment necessitates a comprehensive strategy individualized to factors including age, gender, type, and severity of organ involvement, with a special focus on clinical phenotype.

Interestingly, various therapeutic approaches adopted in PBD management may either mitigate or exacerbate headache symptoms [49]. Firstly, encouraging a balanced diet and physical activity assumes a pivotal role in initial intervention, particularly among younger patients; such an approach could also prevent headache recurrences. Exploring dietary interventions that may also influence the microbiota could offer potential benefits for managing both headaches and PBD outcomes, as can be observed in other diseases [50,51], although additional studies may be required to fully elucidate their efficacy in this context.

Moreover, prevalent manifestations of BD in the pediatric population appear to predominantly involve mucocutaneous presentations. Treating these symptoms usually starts with topical interventions, such as applying local corticosteroids. Additionally, colchicine is generally recommended in patients with erythema nodosum [49], albeit it may precipitate headaches as an adverse effect, whereas azathioprine is recommended for more severe mucocutaneous phenotypes, as well as in phenotypes involving gastrointestinal and neurological symptoms [49]. On the other hand, it must be noted that apremilast, currently under investigation in pediatric PBD cohorts [49], has been closely correlated with headache occurrences according to an analysis conducted by Musialowicz et al. through the FDA Adverse Event Reporting System [52].

A repertoire of pharmacotherapeutic agents including corticosteroids, azathioprine, cyclophosphamide, methotrexate, and TNFα antagonists have been adopted for addressing pNBD [49]. Interestingly, methotrexate has been involved in forms of neurotoxicity, although such manifestations mainly occur in case of high doses and intrathecal administration [53]. Corticosteroids are administered in cases with dural sinus thrombosis, whereas anti-TNF agents may be contemplated in patients with recurrent forms or in cases of arterial involvement [49]. Interestingly, Emmi et al. conducted a retrospective study demonstrating that adalimumab-based regimens exhibited superior and swifter efficacy compared to disease-modifying antirheumatic drugs in facilitating the remission of venous thrombosis among patients with BD, thereby allowing a reduction in steroid administration [54].

Owing to its neurotoxic potential, cyclosporine is contraindicated for individuals suffering from neurological diseases [55]. Moreover, the concurrent administration of NSAIDs during acute headache episodes may exacerbate cyclosporine-associated nephrotoxic sequelae, particularly in the presence of dehydration [56,57].

In the management of primary headaches in patients with BD, acute episodes can be treated with NSAIDs, with careful monitoring for potential interactions with previously mentioned BD therapies. Preventive treatment begins with measures similar to those used in the general population. Since headaches can accompany BD, it is important to avoid other triggers and to address sleep quality, which can be disturbed in this patient group [58]. Regarding pharmacotherapy, it does not appear to be any specific contraindication to the use of medications such as gabapentin, amitriptyline, or topiramate. Notably, topiramate has been successfully used to treat headache associated with pseudotumor cerebri in pediatric NBD [59].

## 4. Conclusions

Headache is a common symptom in pediatric BD, occurring in approximately 25–40% of patients with the disease. Surprisingly, tension-type headache is the most common type of primary headache in BD, whereas there is a lack of studies in the pediatric population; however, assessing TTH in this group could be difficult. On the other hand, headache associated with uveitis is the most common type of secondary headache in PBD. Other types of headaches, such as migraine and cluster headache, can also occur in PBD, although they are less common. Remarkably, pseudotumor cerebri can emerge even as the initial clinical feature of pNBD for numerous patients. Isolated intracranial hypertension should therefore alert to the potential occurrence or development of BD in a pediatric patient, prompting careful monitoring. Treatment of headache in PBD depends on the type of headache and the underlying disease process, with primary headaches often managed with lifestyle modifications and over-the-counter pain relievers, and secondary headaches requiring more aggressive treatment with corticosteroids and other immunosuppressive agents. While pediatric BD is a rare disease, awareness of the potential for headache in these patients can help with timely diagnosis and management. Further research is needed to better understand the underlying mechanisms of headache in PBD and to develop more effective treatment strategies for these patients.

These epidemiological findings not only shed light on the high prevalence and clinical impact of headaches in PBD but also underscore the importance of comprehensive evaluation and management strategies, particularly in the context of associated systemic conditions, in order to optimize clinical outcomes and mitigate long-term sequelae.

## Figures and Tables

**Table 1:** Patients with headache secondary to pNBD; ADEM: acute disseminated encephalomyelitis.

			Prior Diagnosis of BD	Other Symptoms	Imaging	Management
Uluduz et al., 2011 [12]	3	M, 13 years old F, 8 years old F, 15 years old	Yes (2/3) No (1/3)	Hemiparesis, seizures	Encephalomyelitis	
Kara et al., 2005 [32]	1	M, 10 years old	No	Fever, vomiting	Leptomeningeal enhancement of parietal lobes	Methylprednisolone, colchicine, azathioprine, low-dose aspirin
Tocker et al., 2022 [33]	1	F, 16 years old	No	Facial nerve paralysis	Hyperintense T2 right lateral ventricle and periventricular area and temporal region of the pons lesions	Colchicine, steroids
Metreau-Vastel et al., 2010 [34]	2	M, 10 years old M, 15 years old	No	Romboencephalitis, fever	Hyperintense T2 thalamic, brainstem and internal capsule lesions	
Ramachandran et al., 2011 [35]	1	M, 15 years old	Yes	Vomiting, blurred vision	Retinal detachment, hyperintense T2 cortical and midbrain lesions	Methylprednisolone, prednisone, azathioprine
Saltik et al., 2004 [36]	1	M, 14 years old	No	Right hemiparesis	Hyperintense T2 lesions, pontine and thalamic capsule lesions	
Lackmann et al., 2004 [37]	1	F, 15 years old	No	Vomiting, fever, gait disturbances	ADEM	Steroids, azathioprine ibuprofen
Mitra et al., 1999 [38]	1	F, 10 years old	No	Blurred vision	Aseptic meningitis	
Vignola et al., 2001 [39]	1	M, 9 years old	No	N/A	Aseptic meningitis	
Hatachi et al., 2006 [40]	1	M, 10 years old	Yes	Fever, vertigo, hearing loss	Aseptic meningitis	

**Table 2:** Patients with headache secondary to non-parenchymal NBD; CVST: Cerebral Venous Sinus Thrombosis; LMWH: Low-Molecular-Weight Heparin; ASA: Acetylsalicylic Acid; MTX: methotrexate; ASMs: Anti-seizure Medicaments.

			Prior Diagnosis of BD	Other Symptoms Associated with Headache	Imaging	Management
Uluduz et al., 2011 [12]	23	21 M, aged 10–16 years old 2 F, 10–14 years old	Yes (22/23) No (1/23)	N/A	CVST, cranial nerve palsy	
Tocker et al., 2022 [33]	1	M, 15 years old	Yes	Vertigo	Papilledema	Colchicine
Metreau-Vastel et al., 2010 [34]	5	M, 11 years old F, 6 years old M, 7 years old M, 8 years old F, 15 years old	Yes (3/5) No (2/5)	N/A	CVST	Intravenous or oral steroids, colchicine, LMWH or anti-vitamin K agents, azathioprine
Vignola et al., 2001 [39]	1	M, 8 years old	No	Diplopia	CVST	Steroids, colchicine
Yilmaz et al., 2011 [40]	1	F, 12 years old	No	Nausea, vomiting, diplopia	Transverse and rectus sinus thrombosis	Steroids, colchicine, azathioprine
Cakar et al., 2014 [41]	2	F, 13 years old F, 14 years old F, 14.5 years old	Yes	Vomiting	Isolated intracranial hypertension (2/3), intracranial hypertension and CVST (1/3)	Steroids, colchicine (with enoxaparin inpatient with CVST)
Paniker et al., 2007 [42]	1	M, 12 years old	No	Diplopia, blurred vision	CVST, bilateral papilledema	Heparin, ASMs
Can et al., 2006 [43]	1	M, 12 years old	Yes	Fatigue, blurred vision	CVST	Steroids, colchicine, anti-thrombotic therapy
Chaloupka et al., 2003 [44]	1	M, 13 years old	No	Photophobia	CVST	Prednisone, cyclosporine, coumarin
Budin et al., 2002 [45]	1	M, 9 years old	No	Diplopia	CVST	Steroids, MTX, ASA
Pamir et al., 1981 [46]	1	M, 14 years old	Yes		Isolated intracranial hypertension	
Neudorf et al., 2003 [47]	1	F, 12 years old	Yes		Isolated intracranial hypertension	Steroid therapy and acetazolamide, lumbar punctures
Wu et al., 2023 [48]	1	M, 8 years old	No	Diplopia	Abnormal focal signal changes in both frontal and parietal lobes representing focal demyelination	Methylprednisolone, azathioprine, mannitol, mecobalamin

## Supplementary Materials

The following supporting information can be downloaded at: https://www.mdpi.com/article/10.3390/jcm13133659/s1, Figure S1: Flow chart showing the process of article selection.
