# Supplementary figures and images for: Exploring Headaches in Pediatric Behçet Disease: Prevalence, Clinical Impact, and Management
Source: J Clin Med. 2024 Jun 23;13(13):3659. doi: 10.3390/jcm13133659 (PMC11242365; doi:10.3390/jcm13133659)

**Figure S1 (suppl.).** Flow chart showing the process of articles selection.

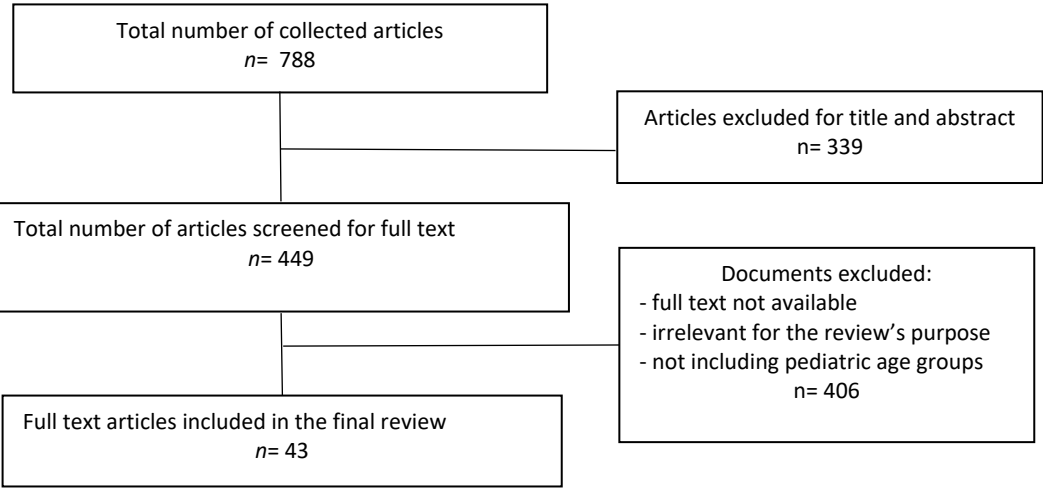

Supplement: Supplementary file 1 [file jcm-13-03659-s001.zip › jcm-3024578-supplementary.pdf]
